# Editorial: Advancements in translational stroke research: spotlight on the blood-brain barrier

**DOI:** 10.3389/fphar.2026.1946057

**Published:** 2026-08-11

**Authors:** Hansen Chen, Chinyere Agbaegbu Iweka, Yi Yang, Dirk M. Hermann

**Affiliations:** 1 Department of Neurosurgery, Stanford University School of Medicine, Stanford, CA, United States; 2 Department of Pharmacology, Case Western Reserve University School of Medicine, Cleveland, OH, United States; 3 Department of Neurology, University of New Mexico, Albuquerque, NM, United States; 4 Department of Neurology, University Hospital Essen, University of Duisburg-Essen, Essen, Germany

**Keywords:** blood–brain barrier, drug delivery, hemorrhagic transformation, lipid metabolism, neural stem cells, secondary prevention, stroke

Reperfusion therapy has redefined acute ischemic stroke care, yet more than half of patients treated with endovascular thrombectomy still do not regain functional independence, reminding us that restoring blood flow does not necessarily guarantee the protection of reperfused brain tissue (Goyal et al., 2016). The reperfusion era has renewed attention to secondary injury mechanisms, including blood–brain barrier (BBB) dysfunction (Candelario-Jalil et al., 2022) and peripheral immune cell infiltration (Shi et al., 2021). Far from a passive conduit, the BBB is an active determinant of edema, hemorrhagic transformation, immune trafficking, and drug access. This Research Topic brings together four contributions that view the barrier from complementary vantage points, spanning ischemic and hemorrhagic stroke and moving from bedside prevention to mechanism, delivery, and repair. Together, these studies reinforce a central concept: the BBB is not merely a structural barrier, but a therapeutic target, a biological obstacle, and a critical determinant of both efficacy and safety.

Two contributions address a question clinicians face daily—how to prevent recurrence after minor stroke or transient ischemic attack (TIA) without provoking hemorrhage. Qin et al. applied a Bayesian network meta-analysis (12 studies; 61,281 patients) to compare single- and dual-antiplatelet regimens. Ticagrelor, a P2Y12 antagonist, combined with aspirin was associated with better efficacy than clopidogrel plus aspirin, including a higher likelihood of excellent functional outcomes (modified Rankin Scale 0–1), and a lower risk of recurrent stroke and major vascular events at 90 days, although these benefits came at the cost of increased bleeding. In contrast, dipyridamole plus aspirin was associated with the lowest bleeding risk among dual antiplatelet regimens, although the supporting evidence was limited and largely indirect. In a meta-analysis of 11 Asian studies including 4,473 patients, Wang et al. reported that dual antiplatelet therapy including the phosphodiesterase-3 inhibitor cilostazol significantly reduced recurrent ischemic stroke (risk ratio [RR], 0.54) and overall stroke recurrence (RR, 0.52) compared with aspirin or clopidogrel monotherapy. Of note, these benefits were not accompanied by a significant increase in serious adverse events, including intracranial hemorrhage. Headache was the most common adverse event, although the excess risk relative to control attenuated with longer follow-up. Clinical benefit was evident at follow-up beyond 3 months. Neither study was designed to investigate the BBB directly. Nevertheless, both highlight a critical clinical challenge in stroke prevention: preventing recurrent ischemic events while minimizing bleeding risk. Hemorrhagic transformation reflects, at least in part, loss of cerebrovascular and BBB integrity, and this risk limits how aggressively antithrombotic therapy can be used. Qin et al. underscore the importance of CYP2C19 loss-of-function status in determining clopidogrel efficacy, whereas Wang et al. emphasize the influence of ethnic and vascular backgrounds on treatment response. The cerebral vasculature critically interacts with activated platelets. Whether and to which degree the vasodilatory and endothelial-protective effects of cilostazol contribute to the favorable stroke prevention profile of the phosphodiesterase-3 inhibitor, remains to be determined. These findings support further investigation of antiplatelet strategies that combine effective ischemic protection with improved vascular safety.

The remaining two contributions move beyond clinical strategies to explore mechanisms of injury, therapeutic delivery, and repair. review how lipid metabolism and lipid droplets regulate neuroinflammation and BBB integrity across neurological diseases, with ischemic stroke as a key example. Their review positions lipid metabolism as an emerging regulator of BBB integrity and neuroinflammation, with lipid peroxidation and ferroptosis providing mechanistic links between metabolic dysregulation and BBB disruption. Notably, they highlight lipid-based nanocarriers as a promising approach for overcoming BBB-related delivery constraints, integrating two overarching themes of this Research Topic: BBB–immune crosstalk and innovative drug delivery. Song et al. extend the discussion to hemorrhagic stroke by reviewing neural stem cell (NSC) therapy for intracerebral hemorrhage. Their review highlights the BBB as both a barrier to therapeutic delivery and a target for repair. While the BBB limits delivery efficiency, motivating targeted and nanomaterial-assisted approaches, NSCs can strengthen neurovascular unit integrity by upregulating tight junction protein expression and reducing barrier leakage, even as the authors caution that cell therapy may transiently worsen permeability during the acute phase, underscoring that BBB modulation is bidirectional and timing-dependent. Together with their synthesis of delivery routes, preclinical models, and early-phase trials, the review highlights both the potential and the challenges of regenerative therapies for hemorrhagic stroke.

Overall, these studies reflect the recent growing interest in BBB biology in stroke, ranging from efforts to preserve BBB integrity and prevent stroke recurrence and hemorrhagic transformation, to defining mechanisms of BBB injury, and to developing therapeutic strategies to promote repair and recovery. Together, these studies highlight a clear translational gap: while the BBB is increasingly recognized as a critical mediator of stroke injury and recovery, we are still limited in our ability to translate this biology into safe, effective therapies. Future progress will depend not only on reopening blocked vessels, but also on understanding the timing, context, and patient-specific factors that determine when the BBB should be preserved, modulated, or repaired.
